# Supplementary material for: Barriers and facilitators to physical activity: A comparative analysis of transplant athletes competing in high intensity sporting events with other transplant recipients
Source: PLoS One. 2024 Aug 23;19(8):e0307095. doi: 10.1371/journal.pone.0307095 (PMC11343464; doi:10.1371/journal.pone.0307095)
Supplement: S1 File — (DOCX) [file pone.0307095.s001.docx]

**Consolidated criteria for reporting qualitative studies (COREQ): 32-item checklist (1)**

| **No** | **Item** | **Guide questions/description** |
| --- | --- | --- |
| **Domain 1: Research team and reflexivity** | | |
| Personal Characteristics | | |
| 1. | Interviewer/facilitator | BR (Except for the interview with BR, which was conducted by co-author KR) |
| 2. | Credentials | PhD in Educational Psychology |
| 3. | Occupation | Professor in Learning Analytics |
| 4. | Gender | Male |
| 5. | Experience and training | 24 years of academic experience, published over 300 academic outputs, including books and provided trainings on mixed methods approaches. 16 years’ experience as transplant recipient and therefore has extensive lived experience of the target population. |
| Relationship with participants | | |
| 6. | Relationship established | Participants were fellow transplant athletes who competed at World Transplant Games (WTG) 2023 in Perth, Australia |
| 7. | Participant knowledge of the interviewer | Participants were informed about the purpose of the study (https://sites.google.com/view/cycling-triathlon-wtg-perth/home/study-1-wtg-2023). Most participants would have met the interviewer at WTG. |
| 8. | Interviewer characteristics | As part of the PPI approach, all the data was sent back to interviewees for further commenting. Furthermore, two unrelated researchers ED (female, senior lecturer in sports psychology) and LP (female, senior research manager transplantation centre), both unrelated to the research participants, and without a role in initial data collection, independently analysed the complete transcripts of three participants using the coding approach adopted by BR. |
| **Domain 2: study design** | | |
| Theoretical framework | | |
| 9. | Methodological orientation and Theory | Mixed methods including content analysis of interview data and race performance data |
| Participant selection | | |
| 10. | Sampling | In total 121 unique TxA from 27 countries were identified for inclusion who participated in cycling and/or triathlon during the WTG 2023. Using purposeful sampling and stratification techniques, potential participants were selected based on their gender, age cohort, country, and relative performance during the cycling and/or sprint triathlon. |
| 11. | Method of approach | Participants were recruited in two ways. Firstly, 28 participants were approached directly in July 2023, out of which 22 (79% response rate) were subsequently interviewed. First author BR is a TxA in cycling and triathlon at WTG, and therefore has a social connection with many of these participants via social media and Online Social Fitness Networks (OSFN), notably Strava (13). Secondly, social media platforms, including the official WTG website and several Strava and Facebook groups (e.g., Transplant Cyclists of the World, GB Transplant cyclists), were used to post an open invitation for anyone who competed in the cycling/triathlon WTG 2023 events to participate in the interview in July-August 2023. Five additional participants were recruited who were not initially sampled. |
| 12. | Sample size | 27 |
| 13. | Non-participation | 1 potential participant dropped out due to a scheduling conflict. |
| Setting | | |
| 14. | Setting of data collection | The race data was collected at Perth, Australia. The interviews were conducted online. |
| 15. | Presence of non-participants | No |
| 16. | Description of sample | In order to ensure anonymisation we classified participants based on continent of origin and rounded the best position in their respective races based on a factor of 10 (e.g., P6 was in the Top10 of best performing TxA, while P20 was in the Top30). Participants were from nine countries across four continents (18 Europe, 6 Australasia, 2 North America, 1 Africa). Of the 27 participants who were interviewed, four (14%) identified as female TxA and 23 (86%) identified as male TxA. 96% participated in cycling, 37% participated in sprint triathlon, and 33% participated in both disciplines |
| Data collection | | |
| 17. | Interview guide | See above. The interview schedule was pilot tested on three authors who participated in the WTG 2023, which indicated that the structure worked well. |
| 18. | Repeat interviews | No |
| 19. | Audio/visual recording | Yes the online interview was audio and visual recorded in MS Teams, but only the audio transcripts were subsequently used. As part of the visual artefact exercise in question 19, we visually illustrated the Table 3 of Adrichem et al. (2016) study in order to prompt participants, and how their experiences resonated with those from participants in that study. |
| 20. | Field notes | Yes the interviewer kept notes for each interview. |
| 21. | Duration | Interviews lasted between 45 and 60 minutes (Mean = 54:44 minutes, SD = 11:04; Range 28:48 -1:06:45) |
| 22. | Data saturation | Yes after around 20 interviews we reached saturation. However, as the remaining participants were keen to participate we also interviewed them. |
| 23. | Transcripts returned | All 27 transcripts were sent back to participants for sense checking, and participants could add further information if needed (five participants did). |
| **Domain 3: analysis and findings** | | |
| Data analysis |  |  |
| 24. | Number of data coders | Three. BR coded all 27 interviews in their entirety to pick up on barriers and facilitators conceptualised by Van Adrichem et al. (5) and raised organically by participants in the first three parts of the interview and in the fourth part of the interview when participants were asked specifically to reflect on barriers and facilitators using the visual artefact. Subsequently, co-authors ED and LP, both unrelated to the research participants and without a role in initial data collection, independently analysed the complete transcripts of three participants using the coding approach adopted by BR. As the inter-rater reliability with one coder was initially relatively low, a follow-up online meeting was arranged with the three coders to clarify the procedure of coding. The final inter-rater reliability between the three coders ranged between 0.72 - 0.83 (Cohen’s Kappa), indicating substantial agreement between the coders |
| 25. | Description of the coding tree | Yes and these followed the structure provided by Van Adrichem et al. (5) |
| 26. | Derivation of themes | Both. The starting point were the codes from Van Adrichem et al. (5), but new codes were added based upon the data (e.g., joy of competition) |
| 27. | Software | Nvivo 12 |
| 28. | Participant checking | Yes both the transcripts as well as the first draft of the paper was sent to all 27 participants for commenting. |
| Reporting |  |  |
| 29. | Quotations presented | Yes, and each quotation included participant number, gender, type of transplant, age group, time of quote in interview, and race performance. |
| 30. | Data and findings consistent | For each barrier and facilitator to PA we compared the results of our study with those of Van Adrichem et al. (5). |
| 31. | Clarity of major themes | See previous |
| 32. | Clarity of minor themes | Some participants reported different experiences to the majority of TxA, which were included both quantitatively and qualitatively. |

1. Tong A, Sainsbury P, Craig J. Consolidated criteria for reporting qualitative research (COREQ): a 32-item checklist for interviews and focus groups. International Journal for Quality in Health Care. 2007;19(6):349-57. doi: 10.1093/intqhc/mzm042.
