# Supplementary material for: Barriers and facilitators to physical activity: A comparative analysis of transplant athletes competing in high intensity sporting events with other transplant recipients
Source: PLoS One. 2024 Aug 23;19(8):e0307095. doi: 10.1371/journal.pone.0307095 (PMC11343464; doi:10.1371/journal.pone.0307095)
Supplement: S3 File — (DOCX) [file pone.0307095.s003.docx]

**Indicative semi-structure interview questions**^^[[1]](#footnote-1)^^

1. Warming up/informal conversation
2. Explanation goal interview and introduction interviewer
3. Informed consent (audio taping and use of data including Garmin/Strava - purpose of this training data explained)
4. Explanation definition of physical activity and high-intensity sport

*Semi-structured interview*

1. Why did you participate in the World Transplant Games in Perth in cycling and/or sprint triathlon?
   1. What is your motivation? (e.g., medals, achievement, post-transplant recovery)^^[[2]](#footnote-2)^^
2. Can you tell me about your transplant journey?
   1. When was it?
   2. How much notice did you have?
   3. What was your recovery like?
3. To what extent were you and your support network (e.g., family, friends, medical and social care professionals) able to prepare for life after your transplant?
   1. e.g. what advice did you seek and what measures, if any, did you take in preparation?
4. How would you describe your fitness capacity before your transplant?
   1. How physically active were you in a typical week?
   2. e.g. job/commuting activities/ sports/ occupational activities
   3. Go deeper into volume of training (weekly mileage) if applicable.
5. Can you describe how physically active you are NOW during a typical week?
   1. e.g. job/commuting activities/ sports/ occupational activities
   2. Go deeper into volume of training (weekly mileage) if applicable.
   3. years of experience in respective sport, discipline, and riding purpose ([Lensing, 2022](#_heading=h.2xcytpi); [Priego Quesada et al., 2018](#_heading=h.2bn6wsx); [Rivers, 2020](#_heading=h.qsh70q))
6. Are you satisfied with your current activity level?
   1. And if not, what would you like to change?
7. Would you consider yourself a competitive athlete?
   1. Do you participate to win a medal, or to complete the challenge?
8. Can you talk about how being a transplant athlete is part of your identity?
   1. How is any athletic identity balanced with other parts of your social, family and work life
9. Do you experience barriers to being physically active or to becoming more physically active?
   1. If so, what are they? ([van Adrichem et al., 2016](#_heading=h.ihv636))
10. Were there any restrictions given by the medical doctor or the physical therapist in what you could do after your transplantation? ([van Adrichem et al., 2016](#_heading=h.ihv636))
11. Do you experience anxiety or restraint to perform certain activities, and if so, which activities and why? ([van Adrichem et al., 2016](#_heading=h.ihv636))
12. In what way have your family and/or close friends (if applicable) had an influence in your life
    1. Before your transplant?
    2. During your transplant?
    3. After your transplant?
13. Do you think that your family and/or close friends (if applicable) have had an influence on being physically active and participating in the World Transplant Games?
14. Who or what has had the largest influence on you being physically active and participating in the World Transplant Games? (e.g., your doctor, your partner, your best friend, your donor, etc.) Why?
15. Finally, we would like to give you a visual artefact of a study conducted amongst 16 transplant recipients in the Netherlands who were asked about the facilitator and barriers to physical activity. In their study [van Adrichem et al. (2016)](#_heading=h.ihv636) found several personal and environmental factors that influenced whether or not transplant recipients would continue with physical exercise or not. We already talked about many of these factors during your interview, such as X, Y, and Z. In your opinion are there any barriers or facilitators that particularly resonate with you?


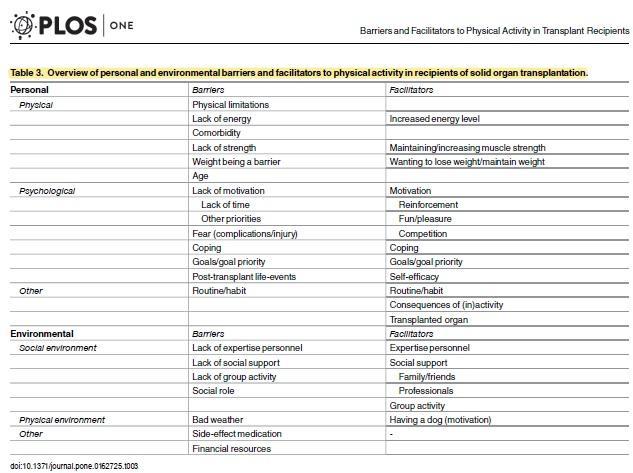


- 1. Is there anything missing from this list?

*Closing question*

1. Do you want to add something or did we miss something you would like to mention related to the topic of the interview?

1. Based upon (adjusted) interview structure of van Adrichem et al. (2016) [↑](#footnote-ref-1)
2. Probing prompts throughout the interview: Can you give an example? Could you explain that in more detail? How? [↑](#footnote-ref-2)
