# Supplementary material for: Barriers and facilitators to physical activity: A comparative analysis of transplant athletes competing in high intensity sporting events with other transplant recipients
Source: PLoS One. 2024 Aug 23;19(8):e0307095. doi: 10.1371/journal.pone.0307095 (PMC11343464; doi:10.1371/journal.pone.0307095)
Supplement: Attachment — Submitted filename: Response to Reviewer 15 11 2023.docx [file pone.0307095.s004.docx]

Response to Reviewer

Hereby, we would like to offer our manuscript entitled “Barriers and facilitators to physical activity: a comparative analysis of transplant athletes competing in high intensity sporting events with other transplant recipients” to your journal *PLOS ONE*. This is a new submission following reviewers comments on a previous submission of this work to *PLOSONE* coded PONE-D-23-36337.

We thank the editorial board and reviewers for feedback regarding PONE-D-23-36337 and in this new submission have sought to address such feedback. Specifically, we addressed the first round of feedback from an email dated 6/11/2023. In that the request was to ensure we completed Human Participants Research Checklist, which we have now included in our current submission. We were also asked to provide additional details regarding participant consent and in particular what type we obtained (for instance, written or verbal, and if verbal, how it was documented and witnessed) and to clarify that our study did not include minors as well as matters around waiving of consent. We can confirm that our study did not include minors, with the participant pool being adults from age bracket 18-29 through to 60-69. Consent was both verbal and written and participants were informed that they could withdraw from the study at any point. Written consent was also followed up by verbal consent at the point of the interview component of our study. Participants were aware of informed consent re the recording of interviews prior to them beginning.

To address a second email dated 13/11/2023 suggesting we had breached the Declaration of Helsinki we clarified by email (sent 13/11/2023) that none of the authors and / or researchers connected to submission coded PONE-D-23-36337 and its research were part of the ethics review board prompting us to ask for a reinstatement of our submission due to a misunderstanding around names of those involved on the ethical review and in the research itself. Here, again we would like to allay fears by re-iterating the contents of that email. That is that a Dr Duncan Banks (of the Open University) was part of the review panel but there was nor is no direct or indirect relation between Dr Duncan Banks, Prof Bart Rienties and / or Dr Elaine Duncan, and none of the authors of the submission have been involved in the ethical review board viewing of or decision to approve this research.

We hope we have now addressed all matters pertaining from the review of PONE-D-23-36337 and now present a fresh submission for consideration.

Thank you
