# Supplementary material for: Barriers and facilitators to physical activity: A comparative analysis of transplant athletes competing in high intensity sporting events with other transplant recipients
Source: PLoS One. 2024 Aug 23;19(8):e0307095. doi: 10.1371/journal.pone.0307095 (PMC11343464; doi:10.1371/journal.pone.0307095)
Supplement: Attachment — Submitted filename: Review comments.doc [file pone.0307095.s005.doc]

## Review comments on manuscript entitled "The effect of six weeks of mirror training program on hand-grip strength and quality of life in children with Spastic Hemiplegic Cerebral Palsy"

**Comments to the Author**

Thank you for the opportunity to review this study. The idea of research is very interesting, organized and well written reasonable. The authors have done great effort to accomplish this work. However, there are some comments and suggestions.

**Section-by-section comments**

**Title:** Well structured, concise and informative.

**Abstract**:

- It is recommended to write keywords in alphabetical order.

**Methodology**:

- What do you mean with [10 mins of conventional therapy], mention the exercises.
- It is better to the details of each test in this section rather than introduction. Including the test validity, reliability, scoring and interpretation.

**Statistical analysis:**

- Well structured

**Discussion:**

- Well structured
