# Supplementary material for: Barriers and facilitators to physical activity: A comparative analysis of transplant athletes competing in high intensity sporting events with other transplant recipients
Source: PLoS One. 2024 Aug 23;19(8):e0307095. doi: 10.1371/journal.pone.0307095 (PMC11343464; doi:10.1371/journal.pone.0307095)
Supplement: Attachment — Submitted filename: Response to Reviewers.docx [file pone.0307095.s006.docx]

Dear reviewers, dear Dr Jianhong Zhou,

We would like to thank the editor and reviewers for their constructive, in-depth and detailed reviews. We have given careful consideration to the issues raised by the Editor and the reviewers and we have revised the article accordingly. We have responded to all the matters raised by the reviewers on a point-by-point basis and these are made clear in the subsequent pages of this letter. We have also highlighted major changes in track changes in the revised manuscript, while minor changes (e.g., grammar, typos) are not highlighted to enhance the readability of the manuscript.

Our article has benefited greatly from this revision process and we would like to thank you and both reviewers for your constructive engagement with our work. We have made every effort to meet the requirements specified by you and the reviewers. We very much hope that, in doing so, we have been able to address the issues raised in effective ways.

We look forward to hearing from you.

Kind regards,

Author A-F.

| 1 | **Reviewer Comments to the Author**  PONE-D-23-39099  Barriers and facilitators to physical activity: a comparative analysis of transplant athletes competing in high intensity sporting events with other transplant recipients.  PLOS ONE  Dear [Author blinded],  Thank you for submitting your manuscript to PLOS ONE. After careful consideration, we feel that it has merit but does not fully meet PLOS ONE’s publication criteria as it currently stands. Therefore, we invite you to submit a revised version of the manuscript that addresses the points raised during the review process.  Please submit your revised manuscript by Jun 21 2024 11:59PM. If you will need more time than this to complete your revisions, please reply to this message or contact the journal office at plosone@plos.org. When you're ready to submit your revision, log on to https://www.editorialmanager.com/pone/ and select the 'Submissions Needing Revision' folder to locate your manuscript file.  Please include the following items when submitting your revised manuscript:  • A rebuttal letter that responds to each point raised by the academic editor and reviewer(s). You should upload this letter as a separate file labeled 'Response to Reviewers'.  • A marked-up copy of your manuscript that highlights changes made to the original version. You should upload this as a separate file labeled 'Revised Manuscript with Track Changes'.  • An unmarked version of your revised paper without tracked changes. You should upload this as a separate file labeled 'Manuscript'.  If you would like to make changes to your financial disclosure, please include your updated statement in your cover letter. Guidelines for resubmitting your figure files are available below the reviewer comments at the end of this letter.  If applicable, we recommend that you deposit your laboratory protocols in protocols.io to enhance the reproducibility of your results. Protocols.io assigns your protocol its own identifier (DOI) so that it can be cited independently in the future. For instructions see: https://journals.plos.org/plosone/s/submission-guidelines#loc-laboratory-protocols. Additionally, PLOS ONE offers an option for publishing peer-reviewed Lab Protocol articles, which describe protocols hosted on protocols.io. Read more information on sharing protocols at https://plos.org/protocols?utm_medium=editorial-email&utm_source=authorletters&utm_campaign=protocols.  We look forward to receiving your revised manuscript.    Kind regards,  Jianhong Zhou  Staff Editor  PLOS ONE  Journal requirements: | **The Authors’ responses**  We are very grateful for the detailed feedback from the editor and the two reviewers. |
| --- | --- | --- |
| 2 | When submitting your revision, we need you to address these additional requirements.  1. Please ensure that your manuscript meets PLOS ONE's style requirements, including those for file naming. The PLOS ONE style templates can be found at  https://journals.plos.org/plosone/s/file?id=wjVg/PLOSOne_formatting_sample_main_body.pdf and  https://journals.plos.org/plosone/s/file?id=ba62/PLOSOne_formatting_sample_title_authors_affiliations.pdf | Thanks for this. We have again checked the guidelines and have ensured that we used all the correct templates and file names. |
| 3 | 2. Please provide a complete Data Availability Statement in the submission form, ensuring you include all necessary access information or a reason for why you are unable to make your data freely accessible. If your research concerns only data provided within your submission, please write "All data are in the manuscript and/or supporting information files" as your Data Availability Statement. | Thanks for this. We have addressed this as follows:  “Interview data cannot be shared publicly because of the interviews contain very sensitive and private information about participants' medical, physical and mental transitions before/during/after transplantation. However, the anonymised quantitative data of the reported barriers and facilitators to Physical exercise per participant are available at the Open University Institutional Data (ORDO) at 10.21954/ou.rd.25878418. Access for researchers who meet the criteria for access to confidential data, including redacted transcripts from three interviews.” |
| 4 | 3. Please remove your figures from within your manuscript file, leaving only the individual TIFF/EPS image files, uploaded separately. These will be automatically included in the reviewers’ PDF. | Ok done |
| 5 | [Note: HTML markup is below. Please do not edit.]  Reviewers' comments:  Reviewer's Responses to Questions  Comments to the Author  1. Is the manuscript technically sound, and do the data support the conclusions?  The manuscript must describe a technically sound piece of scientific research with data that supports the conclusions. Experiments must have been conducted rigorously, with appropriate controls, replication, and sample sizes. The conclusions must be drawn appropriately based on the data presented.  Reviewer #1: Yes  Reviewer #2: Yes | Thanks |
| 6 | 2. Has the statistical analysis been performed appropriately and rigorously?    Reviewer #1: Yes  Reviewer #2: Yes | Ok |
| 7 | 3. Have the authors made all data underlying the findings in their manuscript fully available?  The PLOS Data policy requires authors to make all data underlying the findings described in their manuscript fully available without restriction, with rare exception (please refer to the Data Availability Statement in the manuscript PDF file). The data should be provided as part of the manuscript or its supporting information, or deposited to a public repository. For example, in addition to summary statistics, the data points behind means, medians and variance measures should be available. If there are restrictions on publicly sharing data—e.g. participant privacy or use of data from a third party—those must be specified.    Reviewer #1: Yes  Reviewer #2: Yes | Ok. See also point 3 |
| 8 | 4. Is the manuscript presented in an intelligible fashion and written in standard English?  PLOS ONE does not copyedit accepted manuscripts, so the language in submitted articles must be clear, correct, and unambiguous. Any typographical or grammatical errors should be corrected at revision, so please note any specific errors here.    Reviewer #1: Yes  Reviewer #2: Yes | Ok |
| 9 | 5. Review Comments to the Author  Please use the space provided to explain your answers to the questions above. You may also include additional comments for the author, including concerns about dual publication, research ethics, or publication ethics. (Please upload your review as an attachment if it exceeds 20,000 characters)    Reviewer #1: Your manuscript presents an insightful mixed-methods study comparing transplant athletes with average transplant recipients regarding physical activity barriers and facilitators. This work contributes to the understanding of physical activity in transplant recipients, a vital area of research. However, I have identified several areas that could be strengthened to enhance the clarity, rigor, and impact of your study. | We are very grateful for the detailed feedback from Reviewer 1. |
| 10 | Statistical Analysis and Reporting:  while the statistical methods are standard and the coding process for qualitative data appears robust, the manuscript could be strengthened by providing more detail on the checking of statistical assumptions, explaining the rationale behind the sample size, and including effect sizes and confidence intervals. Additionally, normality and homogeneity of variances. | Thanks for this. In terms of sample size, see point 11. As requested we checked for normality of all reported variables. Given the small sample size and the occasionally skewed responses (e.g., 100% of TxA indicated that PA was a routine/motivation, 0% of TxA indicated comorbidity issues), we redid all the statistical analysis using Mann Whitney U Tests, and reported the effect sizes. The results remained mainly the same, but we have reported the results throughout using z-scores and r in terms of effect size. We have added this as limitation. |
| 11 | It's not clear whether a power analysis was conducted to determine the sample size for quantitative comparisons, which raises questions about the study's power to detect meaningful differences. | Thanks for this. To be honest and open to the reviewers, we did not conduct a power analysis beforehand as we were unsure how many transplant athletes across the globe would be willing to participate in our in-depth study. Our primary goal was to reach a comparable number of 16 participants as Van Adrichem et al. (1), which we managed, whereby in total 27 transplant athletes participated. |
| 12 | Generalizability of Findings:  Consider discussing the limitations regarding the generalizability of your findings. While your study provides valuable insights, the specific nature of your sample may limit its applicability to other transplant recipient populations. | We could not agree more. Also the recent study of (2) caution for overgeneralisation of physical abilities of what transplant recipients can achieve and maintain in the long-term. Therefore, we had added the following in the discussion and conclusion:  “Furthermore, these findings might not be generalisable across a wider group of TxA, or transplant recipients in general. Indeed, (20) warn that the potential affordances and abilities of transplant athletes to compete with high levels of PA might not necessarily translate to a wider and more diverse group of transplant recipients. Furthermore, with a lack of longitudinal studies on the long-term impact of high-intensity levels of PA on health (20) “emphasise that while exercise can serve as a potent therapeutic intervention after transplantation and is probably underutilized in the transplant population, the line between its medicinal benefits and potential harm lies in the dosage administered”.” |
| 13 | Reviewer #2: Review comments on manuscript entitled "Barriers and facilitators to physical activity: a comparative analysis of transplant athletes competing in high intensity sporting events with other transplant recipients"  Comments to the Author  Thank you for the opportunity to review this study. The idea of research is very interesting, organized and well written reasonable. The authors have done great effort to accomplish this work. However, there are some comments and suggestions.  Section-by-section comments | We are also very grateful for the positive feedback from reviewer 2. |
| 14 | Title: Well structured, concise and informative.  Abstract:  - It is recommended to write the abstract in sections including (background, aim, methods, results, conclusion)  - It is recommended to write 5-7 keywords in alphabetical order. | Ok we have adjusted this accordingly. |
| 15 | Introduction :  - Well structured  Methodology:  - Well structured  Statistical analysis:  - Well structured  Discussion:  - Well structured  ________________________________________ | Ok |
| 16 | 6. PLOS authors have the option to publish the peer review history of their article (what does this mean?). If published, this will include your full peer review and any attached files.  If you choose “no”, your identity will remain anonymous but your review may still be made public.  Do you want your identity to be public for this peer review? For information about this choice, including consent withdrawal, please see our Privacy Policy.    Reviewer #1: No  Reviewer #2: No  ________________________________________ | Ok |
| 17 | [NOTE: If reviewer comments were submitted as an attachment file, they will be attached to this email and accessible via the submission site. Please log into your account, locate the manuscript record, and check for the action link "View Attachments". If this link does not appear, there are no attachment files.]  While revising your submission, please upload your figure files to the Preflight Analysis and Conversion Engine (PACE) digital diagnostic tool, https://pacev2.apexcovantage.com/. PACE helps ensure that figures meet PLOS requirements. To use PACE, you must first register as a user. Registration is free. Then, login and navigate to the UPLOAD tab, where you will find detailed instructions on how to use the tool. If you encounter any issues or have any questions when using PACE, please email PLOS at figures@plos.org. Please note that Supporting Information files do not need this step. | Ok We have checked this and have uploaded everything as indicated. |

References

1.	van Adrichem EJ, van de Zande SC, Dekker R, Verschuuren EAM, Dijkstra PU, van der Schans CP. Perceived Barriers to and Facilitators of Physical Activity in Recipients of Solid Organ Transplantation, a Qualitative Study. PLOS ONE. 2016;11(9):e0162725. doi: 10.1371/journal.pone.0162725.

2.	Stylemans D, Vandecruys M, Leunis S, Engelborghs S, Gargioli D, Monbaliu D, et al. Physical Exercise After Solid Organ Transplantation: A Cautionary Tale. Transplant International. 2024;37. doi: 10.3389/ti.2024.12448.
