# Supplementary material for: Barriers and facilitators to physical activity: A comparative analysis of transplant athletes competing in high intensity sporting events with other transplant recipients
Source: PLoS One. 2024 Aug 23;19(8):e0307095. doi: 10.1371/journal.pone.0307095 (PMC11343464; doi:10.1371/journal.pone.0307095)
Supplement: Attachment — Submitted filename: comments.doc [file pone.0307095.s007.doc]

**Review comments on Manuscript Number: (PONE-D-23-39099R1) entitled ‘’ Barriers and facilitators to physical activity: a comparative analysis of transplant athletes competing in high intensity sporting events with other transplant recipients''.**

Overall, this study provides a novel approach.  The idea of research is very interesting, well written and reasonable. I would like to thank the authors for their successful work to address the reviewers' comments. The authors have done great efforts to accomplish this work. They fulfilled all comments and made necessary changes throughput the manuscript. ***I recommend accepting the manuscript its revised form.***
