# Supplementary material for: Barriers and facilitators to physical activity: A comparative analysis of transplant athletes competing in high intensity sporting events with other transplant recipients
Source: PLoS One. 2024 Aug 23;19(8):e0307095. doi: 10.1371/journal.pone.0307095 (PMC11343464; doi:10.1371/journal.pone.0307095)
Supplement: Attachment — Submitted filename: Response to Reviewers.docx [file pone.0307095.s008.docx]

Dear Dr Heather Macdonald, dear reviewers,

We would like to thank the editor and reviewers for their second round of constructive, in-depth and detailed reviews. We have responded to all the matters raised by the reviewers on a point-by-point basis. We have also highlighted major changes in track changes in the revised manuscript, while minor changes (e.g., grammar, typos) are not highlighted to enhance the readability of the manuscript.

We look forward to hearing from you.

Kind regards,

Author A-F.

| 1 | PONE-D-23-39099R1  Barriers and facilitators to physical activity: a comparative analysis of transplant athletes competing in high intensity sporting events with other transplant recipients.  PLOS ONE  Dear Dr. XXX,  Thank you for submitting your manuscript to PLOS ONE. After careful consideration, we feel that it has merit but does not fully meet PLOS ONE’s publication criteria as it currently stands. Therefore, we invite you to submit a revised version of the manuscript that addresses the points raised during the review process.    Please note that I served as the 2nd reviewer on the resubmission, and as the 2nd academic editor I did not provide feedback on the first submission. In my comments below I noted several areas where the manuscript does not currently meet the publication criteria (e.g., COREQ checklist, data availability, detail required on statistical analysis).  Please submit your revised manuscript by Jul 25 2024 11:59PM. If you will need more time than this to complete your revisions, please reply to this message or contact the journal office at plosone@plos.org. When you're ready to submit your revision, log on to https://www.editorialmanager.com/pone/ and select the 'Submissions Needing Revision' folder to locate your manuscript file.  Please include the following items when submitting your revised manuscript:  • A rebuttal letter that responds to each point raised by the academic editor and reviewer(s). You should upload this letter as a separate file labeled 'Response to Reviewers'.  • A marked-up copy of your manuscript that highlights changes made to the original version. You should upload this as a separate file labeled 'Revised Manuscript with Track Changes'.  • An unmarked version of your revised paper without tracked changes. You should upload this as a separate file labeled 'Manuscript'.  If you would like to make changes to your financial disclosure, please include your updated statement in your cover letter. Guidelines for resubmitting your figure files are available below the reviewer comments at the end of this letter.  If applicable, we recommend that you deposit your laboratory protocols in protocols.io to enhance the reproducibility of your results. Protocols.io assigns your protocol its own identifier (DOI) so that it can be cited independently in the future. For instructions see: https://journals.plos.org/plosone/s/submission-guidelines#loc-laboratory-protocols. Additionally, PLOS ONE offers an option for publishing peer-reviewed Lab Protocol articles, which describe protocols hosted on protocols.io. Read more information on sharing protocols at https://plos.org/protocols?utm_medium=editorial-email&utm_source=authorletters&utm_campaign=protocols.  We look forward to receiving your revised manuscript.  Kind regards,  Heather Macdonald, Ph.D  Academic Editor  PLOS ONE  Additional Editor Comments:  Please note that the first academic editor on this manuscript was not available to handle the revision, so it was transferred to me. I also served as the 2nd reviewer as one of the reviewers on the first submission was not available to review the resubmission.  [Note: HTML markup is below. Please do not edit.] | Thanks for the detailed feedback and support. We also appreciate that you had taken over as academic editor for this manuscript. |
| --- | --- | --- |
| 2 | Reviewers' comments:  Reviewer's Responses to Questions  Comments to the Author  1. If the authors have adequately addressed your comments raised in a previous round of review and you feel that this manuscript is now acceptable for publication, you may indicate that here to bypass the “Comments to the Author” section, enter your conflict of interest statement in the “Confidential to Editor” section, and submit your "Accept" recommendation.  Reviewer #2: All comments have been addressed  Reviewer #3: (No Response) | OK |
| 3 | 2. Is the manuscript technically sound, and do the data support the conclusions?  The manuscript must describe a technically sound piece of scientific research with data that supports the conclusions. Experiments must have been conducted rigorously, with appropriate controls, replication, and sample sizes. The conclusions must be drawn appropriately based on the data presented.  Reviewer #2: Yes  Reviewer #3: Partly | OK |
| 4 | 3. Has the statistical analysis been performed appropriately and rigorously?  Reviewer #2: Yes  Reviewer #3: No | OK |
| 5 | 4. Have the authors made all data underlying the findings in their manuscript fully available?  The PLOS Data policy requires authors to make all data underlying the findings described in their manuscript fully available without restriction, with rare exception (please refer to the Data Availability Statement in the manuscript PDF file). The data should be provided as part of the manuscript or its supporting information, or deposited to a public repository. For example, in addition to summary statistics, the data points behind means, medians and variance measures should be available. If there are restrictions on publicly sharing data—e.g. participant privacy or use of data from a third party—those must be specified.  Reviewer #2: Yes  Reviewer #3: No | OK |
| 6 | 5. Is the manuscript presented in an intelligible fashion and written in standard English?  PLOS ONE does not copyedit accepted manuscripts, so the language in submitted articles must be clear, correct, and unambiguous. Any typographical or grammatical errors should be corrected at revision, so please note any specific errors here.  Reviewer #2: Yes  Reviewer #3: Yes | OK |
| 7 | Please use the space provided to explain your answers to the questions above. You may also include additional comments for the author, including concerns about dual publication, research ethics, or publication ethics. (Please upload your review as an attachment if it exceeds 20,000 characters)  Reviewer #2: Review comments on Manuscript Number: (PONE-D-23-39099R1) entitled ‘’ Barriers and facilitators to physical activity: a comparative analysis of transplant athletes competing in high intensity sporting events with other transplant recipients''.  Overall, this study provides a novel approach. The idea of research is very interesting, well written and reasonable. I would like to thank the authors for their successful work to address the reviewers' comments. The authors have done great efforts to accomplish this work. They fulfilled all comments and made necessary changes throughput the manuscript. I recommend accepting the manuscript its revised form. | Thanks for the detailed feedback. |
| 8 | Reviewer #3: Please note that I did not review the initial submission. This is an interesting mixed methods study of barriers to and facilitators of physical activity in transplant athletes. The manuscript is generally well written and presents interesting findings, but I note some key points that should be addressed. | Also thanks to reviewer 3 for the detailed and positive feedback. |
| 9 | 6. Review Comments to the Author  Please use the space provided to explain your answers to the questions above. You may also include additional comments for the author, including concerns about dual publication, research ethics, or publication ethics. (Please upload your review as an attachment if it exceeds 20,000 characters)  Reviewer #2: Review comments on Manuscript Number: (PONE-D-23-39099R1) entitled ‘’ Barriers and facilitators to physical activity: a comparative analysis of transplant athletes competing in high intensity sporting events with other transplant recipients''.  Overall, this study provides a novel approach. The idea of research is very interesting, well written and reasonable. I would like to thank the authors for their successful work to address the reviewers' comments. The authors have done great efforts to accomplish this work. They fulfilled all comments and made necessary changes throughput the manuscript. I recommend accepting the manuscript its revised form. | Thanks for this. |
| 10 | Reviewer #3: Please note that I did not review the initial submission. This is an interesting mixed methods study of barriers to and facilitators of physical activity in transplant athletes. The manuscript is generally well written and presents interesting findings, but I note some key points that should be addressed.  1. As per PLOS ONE guidelines, please consider including the COREQ checklist (http://journals.plos.org/plosone/s/submission-guidelines#loc-qualitative-research). | Many thanks for this suggestion. We have included the COREQ checklist as an online Appendix 2 |
| 11 | 2. In their response to initial reviews, the authors provided a doi for the quantitative data. Unfortunately, the doi did not work for me and I was unable to access the data. I tried searching for ORDO without luck. Please provide the correct link/doi for the data. | Apologies for this mistake. For some reason after submission of the revised manuscript our librarians had some further questions on the data, and hence made the online data set unavailable. We had not realised that the underlying dataset became unavailable afterwards. This has now been addressed and all the quantitative data is available at:  [https://doi.org/10.21954/ou.rd.c.7279993.v1](https://eur01.safelinks.protection.outlook.com/?url=https%3A%2F%2Fdoi.org%2F10.21954%2Fou.rd.c.7279993.v1&data=05%7C02%7Cbart.rienties%40open.ac.uk%7C5b052a941fa44be3f08508dc8a169c93%7C0e2ed45596af4100bed3a8e5fd981685%7C0%7C0%7C638537076918736545%7CUnknown%7CTWFpbGZsb3d8eyJWIjoiMC4wLjAwMDAiLCJQIjoiV2luMzIiLCJBTiI6Ik1haWwiLCJXVCI6Mn0%3D%7C0%7C%7C%7C&sdata=E%2FPBKpt9buxI1v%2FBJOyuFmNlTzzzBBm%2Fa6fFH3b7IZ0%3D&reserved=0)  Three anonymous transcripts of interviews are made available under embargo (i.e., researchers from universities are able to request access to the data). |
| 12 | 3. Regarding the comparisons with data from study by Van Adrichem et al., I see that descriptive data is available for each participant in the Van Adrichem study, but for the other comparisons (e.g., % that reported physical limitations, among others) - did the authors conduct test to compare proportions? Please clarify and modify the methods accordingly. Please also clarify how effect size was determined. | Thanks for this. As previously mentioned to the reviewers, “As requested we checked for normality of all reported variables. Given the small sample size and the occasionally skewed responses (e.g., 100% of TxA indicated that PA was a routine/motivation, 0% of TxA indicated comorbidity issues), we redid all the statistical analysis using Mann Whitney U Tests, and reported the effect sizes.”. In terms of effect size, we used the following formula: r = z/√N |
| 13 | Is this Also, as per the paragraph on "Participant selection" in the Van Adrichem study, "the exact level of physical activity was not assessed" so it does not seem appropriate to describe that cohort as having average activity levels. | Yes that is a fair point. According to the Van Adrichem study “Participants were eligible if they had a stable organ function, and if they comprehended the Dutch language. A purposive  sampling strategy was employed in which participants were selected who would adequately represent diversity and produce relevant information to the research question (i.e., active vs. inactive). Judgment of suitability was made by the treating physician or physician’s assistant on the basis of their impression and anamneses of their latest contact with the patient in the outpatient setting; the exact level of physical activity was not assessed.”  We have therefore adjusted this in the abstract and elsewhere as “to a group of Dutch transplantees”  “The first aim of this study is to identify perceived barriers and facilitators to PA of TxA and compare these to those of Dutch transplant recipients with mixed levels of physical activity.”  “This group of 16 transplantees included a mix of both active and inactive transplantees, although their actual PA levels were not objectively assessed.” |
| 14 | 4. In the last paragraph of the introduction, the authors mention 2 study aims. Yet, the 2nd aim is not mentioned in the abstract, and I did not see any results presented to support this aim. | Thanks for pointing this out. We have removed the second aim and adjusted the manuscript accordingly. |
| 15 | 5. Figure 1 requires modifications as the axis labels do not accurately describe the data presented - is this meant to be the % of TxA who reported barriers/facilitators? | Thanks for this. We have adjusted this accordingly. Note: n = 27. Numbers in the scatterplot refer to respective participant. Scale from 0 (no barriers out of possible 20; no facilitators out of 16) to 1 (20 out of 20 barriers; 16 out of facilitators) |
| 16 | Additional comments  1. Abstract, Aim: Consider modifying the description of the study population ("physically active transplant recipients") to more accurately describe the group (transplant athletes) as is used elsewhere in the manuscript.  ________________________________________ | Ok |
| 17 | 2. Abstract, Aim: Use past tense - change analyses to analysed. Similarly change the aims in the introduction to past tense (The first aim of this study was to..."). | Ok |
| 18 | 3. What is meant by "visual artefact"? Please consider including the "visual artefact" in the manuscript or as supplemental material. Perhaps it could be included with the interview guide. | Yes this virtual artefact was already included in Online Appendix 1. The visual artefact was Table 3 of the Van Adrichem study, whereby we used animations in Powerpoint to highlight specific sections of that Table to participants. We have clarified this in the main manuscript |
| 19 | 4. Line 63: Change the wording of "...recipients indicated to..." | Ok |
| 20 | 5. Line 83: Gender refers to Gender refers to the socially constructed roles, behaviours, expressions and identities of girls, women, boys, men, and gender diverse people - please clarify how gender was assessed in this study. (https://researchintegrityjournal.biomedcentral.com/articles/10.1186/s41073-016-0007-6) | This is a fair point. At present the WTGF has only two categories of athletes (male/female) in terms of race results, and we used the same distinction. |
| 21 | 6. Line 160: How did the authors determine the training intensity of the TxA that didn't volunteer for an interview? | As the first author is linked on Strava with most of the 121 transplant athletes we obtained their training data from Strava where this was available. Afterwards these data were anonymised and subsequently compared to those 27 who gave explicit permission to use their training data, the linked interview, and racing data. |
| 22 | 7. Line 164: Remove the period after size. | Ok |
| 23 | 8. Lines 452-453: Please provide references to support the statement about the wider transplant community and the adult population in general. | Ok |
| 24 | 9. Lines 466-468: Similar to my comment above, data to support the 2nd aim was not presented in the results. Hours of training are mentioned on line 199, but this doesn't appear to be specific to the time of the WTG. | Yes we have rephrased this as indicated above. |
| 25 | 10. Line 484: Change IronMan to Ironman | Ok |
| 26 | 11. Line 506: Please modify the wording of "...an whether in line..." | Ok |
| 27 | 7. PLOS authors have the option to publish the peer review history of their article (what does this mean?). If published, this will include your full peer review and any attached files.  If you choose “no”, your identity will remain anonymous but your review may still be made public.  Do you want your identity to be public for this peer review? For information about this choice, including consent withdrawal, please see our Privacy Policy.  Reviewer #2: No  Reviewer #3: No | Ok |
