# Supplementary material for: Barriers and facilitators to physical activity: A comparative analysis of transplant athletes competing in high intensity sporting events with other transplant recipients
Source: PLoS One. 2024 Aug 23;19(8):e0307095. doi: 10.1371/journal.pone.0307095 (PMC11343464; doi:10.1371/journal.pone.0307095)
Supplement: Attachment — Submitted filename: Response to Reviewers.docx [file pone.0307095.s009.docx]

Dear Dr Heather Macdonald, dear reviewers,

We would like to thank the editor and Reviewer 3 for their third round of constructive, in-depth and detailed reviews. We have responded to all the matters raised by the reviewers on a point-by-point basis. We have also highlighted major changes in track changes in the revised manuscript, while minor changes (e.g., grammar, typos) are not highlighted to enhance the readability of the manuscript.

We look forward to hearing from you.

Kind regards,

Author A-F.

| 1 | PONE-D-23-39099R2  Barriers and facilitators to physical activity: a comparative analysis of transplant athletes competing in high intensity sporting events with other transplant recipients.  PLOS ONE  Dear Dr. Duncan,  Thank you for submitting your manuscript to PLOS ONE. After careful consideration, we feel that it has merit but does not fully meet PLOS ONE’s publication criteria as it currently stands. Therefore, we invite you to submit a revised version of the manuscript that addresses the points raised during the review process.    Thank you for addressing my comments. I noted a few additional points below that require clarification.  Please submit your revised manuscript by Aug 07 2024 11:59PM. If you will need more time than this to complete your revisions, please reply to this message or contact the journal office at plosone@plos.org. When you're ready to submit your revision, log on to https://www.editorialmanager.com/pone/ and select the 'Submissions Needing Revision' folder to locate your manuscript file.  Please include the following items when submitting your revised manuscript:  • A rebuttal letter that responds to each point raised by the academic editor and reviewer(s). You should upload this letter as a separate file labeled 'Response to Reviewers'.  • A marked-up copy of your manuscript that highlights changes made to the original version. You should upload this as a separate file labeled 'Revised Manuscript with Track Changes'.  • An unmarked version of your revised paper without tracked changes. You should upload this as a separate file labeled 'Manuscript'.  If you would like to make changes to your financial disclosure, please include your updated statement in your cover letter. Guidelines for resubmitting your figure files are available below the reviewer comments at the end of this letter.  If applicable, we recommend that you deposit your laboratory protocols in protocols.io to enhance the reproducibility of your results. Protocols.io assigns your protocol its own identifier (DOI) so that it can be cited independently in the future. For instructions see: https://journals.plos.org/plosone/s/submission-guidelines#loc-laboratory-protocols. Additionally, PLOS ONE offers an option for publishing peer-reviewed Lab Protocol articles, which describe protocols hosted on protocols.io. Read more information on sharing protocols at https://plos.org/protocols?utm_medium=editorial-email&utm_source=authorletters&utm_campaign=protocols.  We look forward to receiving your revised manuscript.  Kind regards,  Heather Macdonald, Ph.D  Academic Editor  PLOS ONE    Journal Requirements:  Please review your reference list to ensure that it is complete and correct. If you have cited papers that have been retracted, please include the rationale for doing so in the manuscript text, or remove these references and replace them with relevant current references. Any changes to the reference list should be mentioned in the rebuttal letter that accompanies your revised manuscript. If you need to cite a retracted article, indicate the article’s retracted status in the References list and also include a citation and full reference for the retraction notice.  [Note: HTML markup is below. Please do not edit.] | Ok |
| --- | --- | --- |
| 2 | Reviewers' comments:  Reviewer's Responses to Questions  Comments to the Author  1. If the authors have adequately addressed your comments raised in a previous round of review and you feel that this manuscript is now acceptable for publication, you may indicate that here to bypass the “Comments to the Author” section, enter your conflict of interest statement in the “Confidential to Editor” section, and submit your "Accept" recommendation.  Reviewer #3: (No Response)  2. Is the manuscript technically sound, and do the data support the conclusions?  The manuscript must describe a technically sound piece of scientific research with data that supports the conclusions. Experiments must have been conducted rigorously, with appropriate controls, replication, and sample sizes. The conclusions must be drawn appropriately based on the data presented.  Reviewer #3: (No Response)  3. Has the statistical analysis been performed appropriately and rigorously?  Reviewer #3: (No Response)  ________________________________________  4. Have the authors made all data underlying the findings in their manuscript fully available?  The PLOS Data policy requires authors to make all data underlying the findings described in their manuscript fully available without restriction, with rare exception (please refer to the Data Availability Statement in the manuscript PDF file). The data should be provided as part of the manuscript or its supporting information, or deposited to a public repository. For example, in addition to summary statistics, the data points behind means, medians and variance measures should be available. If there are restrictions on publicly sharing data—e.g. participant privacy or use of data from a third party—those must be specified.  Reviewer #3: (No Response)  ________________________________________  5. Is the manuscript presented in an intelligible fashion and written in standard English?  PLOS ONE does not copyedit accepted manuscripts, so the language in submitted articles must be clear, correct, and unambiguous. Any typographical or grammatical errors should be corrected at revision, so please note any specific errors here.  Reviewer #3: (No Response)  ________________________________________  6. Review Comments to the Author  Please use the space provided to explain your answers to the questions above. You may also include additional comments for the author, including concerns about dual publication, research ethics, or publication ethics. (Please upload your review as an attachment if it exceeds 20,000 characters) | Ok |
| 3 | Reviewer #3: Thank you for responding to my comments. There remain a few issues to be addressed: | Ok thanks for the detailed feedback Reviewer 3 |
| 4 | 1. Unfortunately, the COREQ checklist was not included with the PDF submission. Please also consider referring to all supplementary files as such in the manuscript rather than using the term "Appendix". | Thanks for this. It was available at the end of in the S3 Online Appendix. However, as suggested by the reviewer we have added S3 COREQ as a supplementary document, and added the interview questions as S5. |
| 5 | 2. Apologies for my confusion, but I am still unclear how the authors obtained the data from the Van Adrichem et al. study that was used to conduct the Mann Whitney U tests. The authors provide the data for the TxA in an excel file - do they have a similar file for the Van Adrichem study? Please clarify in the manuscript. | Thanks for this. As Reviewer 3 is correct that the precise individual data was not made available by the Van Adrichem et al. study, we created a synthetic dataset based upon the available aggregated data from “[S1 Table.](https://journals.plos.org/plosone/article/file?type=supplementary&id=10.1371/journal.pone.0162725.s002) Distribution of mentioned barriers and facilitators across transplant recipient groups”. For example, from this S1 Table it can be concluded that 4 out of 4 heart Tx had physical barriers, and 2 out of 4 had energy level barriers, etc. As MWU analyses non-normal distributions or ordinal data, we created a synthetic dataset whereby we allocated the respective reported barriers and facilitators per 4 transplant groupings exactly as reported in S1 Table.  As we only compared reported barriers and facilitators on an individual variable level (e.g., 12 Van Adrichem respondents out of 16 reported lower energy levels relative to 8 out of 27 WTG participants) rather than across variables it would not matter if say in reality heart tx 3 and heart tx 4 rather than heart tx 1 and heart tx2 in the actual data had energy level barriers, as the proportion on this group and the Van Adrichem cohort on this variable would still be the same. We have made this data available in the supplementary file S4.  In the manuscript, we have added the following sentence:  All available quantitative data were analysed with SPSS 27 using Mann Whitney U Tests for non-parametric testing comparing the 27 TxA with the 16 NL transplantees in Van Adrichem et al. (1). As only aggregated data per transplant recipient group were publicly available in (1) we generated a synthetic dataset matching the exact proportions reported per respective barrier and facilitator. |
| 6 | 3. If a specific question about gender was not asked, please change gender to sex throughout the manuscript, as male/female are categories used to describe sex not gender. | Thanks for this. We have adjusted this. |
| 7 | 4. Regarding the training intensity of the TxA that didn't volunteer - please include the information about Strava in the manuscript. | OK. We have adjusted this as follows: In order to check for potential non-response bias, we compared age, sex and training intensity as measured by publicly available Strava data of the 27 participants with the 94 TxA who did not volunteer for an interview, but no statistically significant differences were found. |
| 8 | 5. Regarding Figure 1 - sorry to harp on this, but I still feel that the figure title could be modified to more clearly reflect what is presented. The figure shows a scatterplot of the proportion of TxA that reported barriers to and facilitators of to PA. I feel it is important to clarify the proportion aspect - it is not a scatterplot of just barriers and facilitators on their own. Also, in the figure, the title at the top is not needed since you have a figure caption. Consider also using different colours or perhaps symbols for the type of transplant. It is not possible to distinguish between the heart and kidney participants, and the bone marrow circles are also difficult to distinguish with the black outline. | Many thanks for this. We have adjusted the Figure as suggested, and furthermore have transformed the data from 0-1 to % to enhance the clarity of the results.   |
| 9 | ________________________________________  7. PLOS authors have the option to publish the peer review history of their article (what does this mean?). If published, this will include your full peer review and any attached files.  If you choose “no”, your identity will remain anonymous but your review may still be made public.  Do you want your identity to be public for this peer review? For information about this choice, including consent withdrawal, please see our Privacy Policy.  Reviewer #3: No  ________________________________________  [NOTE: If reviewer comments were submitted as an attachment file, they will be attached to this email and accessible via the submission site. Please log into your account, locate the manuscript record, and check for the action link "View Attachments". If this link does not appear, there are no attachment files.]  While revising your submission, please upload your figure files to the Preflight Analysis and Conversion Engine (PACE) digital diagnostic tool, https://pacev2.apexcovantage.com/. PACE helps ensure that figures meet PLOS requirements. To use PACE, you must first register as a user. Registration is free. Then, login and navigate to the UPLOAD tab, where you will find detailed instructions on how to use the tool. If you encounter any issues or have any questions when using PACE, please email PLOS at figures@plos.org. Please note that Supporting Information files do not need this step. | Ok. We have updated the figure using PACE. |

1.	van Adrichem EJ, van de Zande SC, Dekker R, Verschuuren EAM, Dijkstra PU, van der Schans CP. Perceived Barriers to and Facilitators of Physical Activity in Recipients of Solid Organ Transplantation, a Qualitative Study. PLOS ONE. 2016;11(9):e0162725. doi: 10.1371/journal.pone.0162725.
